# The processing of spatial frequencies through time in visual word recognition

**DOI:** 10.1038/s41598-024-57219-3

**Published:** 2024-03-19

**Authors:** Clémence Bertrand Pilon, Martin Arguin

**Affiliations:** 1 Department of Psychology, Université de Montréal, C.P. 6128, Succ. Centre-ville, Montréal, QC H3C 3J7 Canada; 2 Centre de recherche de l’Institut universitaire de gériatrie de Montréal, Montreal, QC Canada; 3 Centre interdisciplinaire de recherche sur le cerveau et l’apprentissage (CIRCA), Department of Psychology, Université de Montréal, Montreal, QC Canada

**Keywords:** Neuroscience, Psychology

## Abstract

This study examined the temporal profile of spatial frequency processing in a word reading task in 16 normal adult readers. They had to report the word presented in a 200 ms display using a four-alternative forced-choice task (4AFC). The stimuli were made of an additive combination of the signal (i.e. the target word) and of a visual white noise patch wherein the signal-to-noise ratio varied randomly across stimulus duration. Four spatial frequency conditions were defined for the signal component of the stimulus (bandpass Butterworth filters with center frequencies of 1.2, 2.4, 4.8 and 9.6 cycles per degree). In contrast to the coarse-to-fine theory of visual recognition, the results show that the highest spatial frequency range dominates early processing, with a shift toward lower spatial frequencies at later points during stimulus exposure. This pattern interacted in a complex way with the temporal frequency content of signal-to-noise oscillations. The outcome of individual data patterns classification by a machine learning algorithm according to the corresponding spatial frequency band further shows that the most salient spatial frequency signature is obtained when the time dimension within data patterns is recoded into its Fourier transform.

## Introduction

Visual word recognition is a complex task which is a cornerstone for communication and that most human adults perform very efficiently and with little effort. Its execution involves a wide variety of intricate visual mechanisms as well as access to the separate domain of language processing. Previous studies have indicated that visual word recognition is largely based on our ability to recognize its component letters^1–3^, a process that appears as a crucial interface between vision and language.

With respect to the visual processing involved in word recognition, a crucial stimulus dimension pertains to its spatial frequency (SF) content. For the recognition of individual letters, the literature unanimously demonstrates that SFs in the range between 2 and 4 cycles per letter are those upon which the human visual system crucially relies^4–10^. Similarly, an optimal SF range of about 3 to 6 cycles per degree (cpd) has been reported for the recognition of visually presented words^11,12^.

An interesting feature of SF processing which has been the object of several previous investigations under the impetus of the coarse-to-fine theory of visual recognition^13,14^ concerns the way SF preference evolves through time. According to the coarse-to-fine theory, visual processing initially focuses on low SFs, from which a coarse representation of the stimulus is passed on to the frontal lobes to obtain some approximation of its identity. This information is then fed back to the visual system to guide the processing of higher SFs, from which the exact identity of the item can be established. Substantial empirical support for coarse-to-fine visual processing has been reported using tasks involving the recognition of scenes, objects, and faces in a variety of experimental paradigms.

Perhaps the first empirical study pertaining to the issue is that of Schyns and Oliva^15^, which used hybrid stimuli made by the superposition by transparency of the image of a low SF filtered scene (2 cpd) with that of another scene that had been high SF filtered (6 cpd). Their results show a bias towards recognizing the low SF filtered scene with brief exposure durations whereas longer durations were associated with a bias for the high SF scene. The authors concluded that these findings imply coarse-to-fine visual processing. Other studies that have similarly used hybrid stimuli with congruent results are those of Oliva and Schyns^16^ with letters, Morrison and Schyns^17^ with objects, Kauffmann, Roux-Sibilon, Beffara et al.^18^ with scenes, and Wang et al.^19^ with emotional faces.

An alternative approach to investigate the issue is to use bandpass filtered stimuli of various SF ranges and examine performance according to whether the SF content varies from low-to-high or high-to-low across the target duration. An instance of this method is the study of Kauffmann, Chauvin, Guyader & Peyrin^20^, who used a task of rapid scene categorization and stimuli that were spatially filtered to central frequencies of 1.0, 1.4, 2.0, 2.9, 4, 4.2, or 6.0 cpd using bandpass filters with a standard deviation of 1.07 cpd. They report shorter response times in the so called coarse-to-fine condition (i.e. SF content going from low to high), which was expected under the assumption that low SF information becomes available earlier than high SFs in scene categorization. Similar findings were obtained in other studies using this technique^18^, with scenes^21^, with digits). In the context of an fMRI study using a similar stimulation method, Peyrin, Schwartz, Seghier et al.^22^ reported evidence congruent with coarse-to-fine processing in the right occipito-temporal cortex but the opposite processing sequence in corresponding regions of the left cerebral hemisphere.

Yet another method by which the issue of coarse-to-fine visual processing may be investigated is to use stimuli with an SF content that varies randomly throughout exposure to then construct classification images (CIs) reflecting the correlation between processing efficiency and SFs as a function of time since the beginning of stimulus exposure. Among others, this method was used by Caplette, Wicker & Gosselin^23^ in a study involving object recognition. Their findings from normal observers indicate that low SFs (below 20 cycles per image) were the most useful for accurate recognition. However, high SFs were increasingly useful as target exposure progressed, which was interpreted as supporting the coarse-to-fine hypothesis. Other studies using a similar method are those of Caplette, Wicker, Gosselin & West^24^ and Caplette, Gosselin & West^25^ with objects,Wiesmann, Caplette, Willenbockel et al.^26^ with scenes. Again, these studies report observations in support of coarse-to-fine visual processing.

An important issue that may be noted regarding the literature cited above on coarse-to-fine processing is that they used rather peculiar stimuli that diverge in major ways from our daily experience and for which the human visual system may be poorly adapted. Thus, the hybrid stimuli used in a subset of these investigations are images comprising a double identity, those of the low and high SF items. It cannot be excluded that such self-conflicting images may trigger processing mechanisms that are normally not involved in visual recognition. With respect to the other experiments which used images with an SF content that evolved either continuously or randomly through time, we again have a stimulation modality that is quite unusual, with a fundamental image property (i.e. SF content) that is unstable through time. Although we know of no actual evidence showing that either stimulation modality is bound to produce artifactual results, it seems indicated to investigate coarse-to-fine visual processing using an alternative method, in the spirit of converging operations^27^.

Another notable issue pertaining to the above studies is that none of them examined the notion of coarse-to-fine processing in the context of a word recognition task. The only such study of which we are aware is that of Winsler, Holcomb, Midgley & Grainger^28^. They assessed the effect on event-related potentials (ERPs) of lowercase broadband, low-pass (< 3.7 cpd) or high-pass (> 15.2 cpd) 50-ms masked primes on the processing of a subsequent broadband uppercase target word. The priming effects with high-pass primes roughly resembled those with broadband primes whereas those with low-pass primes failed to show any previously documented ERP repetition priming effect. The authors conclude that word recognition fails to replicate the coarse-to-fine effects that have been reported with other stimulus classes. They suggest that this may relate to the fact that “words fundamentally require more precise and complex processing than most other categories of visual stimuli” (p. 11).

The aim of the present study was to assess the time course of SF processing in the specific context of a visual word recognition task. On any given trial, the target word was spatially filtered according to one of four SF bands (retinal spatial frequencies of 1.2, 2.4, 4.8 or 9.6 cpd; object spatial frequencies of 1.7, 3.5, 7.1 or 14.1 cycles per letter – cpl) and remained thus for the entire exposure duration of 200 ms. However, the target was superimposed with a white noise mask and the image displayed was an additive combination of both, with a signal-to-noise ratio (SNR) that varied randomly through time. CIs of processing efficiency as a function of time were then calculated to illustrate how the processing of each SF band evolves through time. According to the coarse-to-fine theory of visual recognition, low SFs should dominate early processing whereas the processing of high SFs should gain in relative efficiency at later periods of stimulus exposure. This observation should however interact with the range of SFs that has previously been found to be optimal for word recognition, such that our two conditions of intermediate SFs are likely to dominate over the more extreme conditions of highest and lowest SFs ranges regardless of time during exposure.

## Results

Response accuracy was very close to 50% correct for all conditions (Table 1), which did not differ from one another (F(3, 45) < 1). The contrast of targets required to reach these accuracies differed significantly across conditions (Table 1; F(3, 45) = 17.6; *p* < 0.001). Thus, the lowest and highest SF conditions required a higher target contrast than the two intermediate conditions, which broadly correspond to SFs in the range that has previously been found optimal for reading (see “Introduction”).

**Table 1:** Response accuracy (in % correct) and levels of target contrast for each SF condition.

SF condition	Accuracy (%)	Contrast (%)
1.2 cpd/1.7 cpl	50.2	18.5
2.4 cpd/3.5 cpl	52.1	12.4
4.8 cpd/7.1 cpl	50.6	13.1
9.6 cpd/14.1 cpl	50.0	18.1

Figure 3 shows the CIs of processing efficiency as a function of time for each SF condition. Strikingly, if we follow the SF range with the greatest level of efficiency from target onset to offset, the order is condition 4 (highest SF range), followed by 3 (second highest SF range), each for a 33 ms duration. Only then, starting at 67 ms does condition 1 (lowest SF range) lead to the greatest efficiency until 150 ms, which is followed by condition 2 (second highest SF range) and then by a brief reprieve of condition 1 on the last display frame. This pattern of results is almost directly opposite to that expected on the basis of the coarse-to-fine theory.

The time-domain CIs from individual participants were submitted to an SVM classifier (with leave-one-out cross validation) which had the task of deciding the SF condition which had produced the set of CI features it was exposed to. The purpose of this procedure was threefold. One was to assess the magnitude of the differences across conditions in the temporal profiles of processing efficiencies illustrated in Fig. 3 and another was to assess their reliability across participants. This last aspect is especially critical given that the Pixel test (used to assess the significance of points in the CIs, see “Methods”^29^) completely ignores whether the patterns of results are replicable across participants. Thus, given sufficiently large differences across the mean patterns of the SF conditions and a good degree of replicability of these patterns across individual participants, then the SVM should attain a high level of accuracy. Given such an occurrence, then we would be able to follow up with the third purpose of using the SVM classifier, which is to illustrate the features of processing efficiency that characterize each condition.

Exposed to features from the time-domain CIs, the SVM classifier reached a maximum response accuracy of 42.2%, which is highly significant against the chance level performance of 25% (binomial test *p* < 0.005). Most impressively, the SVM needed exposure to only three features (i.e. 12.5%) from the 24 available in the time-domain CIs. These features correspond to the processing efficiency values at time frames 3, 7, and 14 (25, 58 and 117 ms, respectively). These correspond to time periods when SF conditions 4, 3, and 1, respectively, offered the best processing efficiency.

In other studies from our lab using random temporal sampling^1,30,31^, classifier performance was typically substantially improved when the data patterns exposed to the SVM pertained not to the CIs themselves, but rather to their Fourier transforms (i.e. phase x power). We will return to this issue in the Discussion section. Given this previous experience, we replicated the SVM classification task, but this time using features that were extracted from the Fourier transforms of the individual time-domain CIs. This led to an improved performance from the classifier, with a maximum classification accuracy of 56.3% correct (significantly superior to the 25% chance performance,binomial test *p* < 0.001) while using 58 features (i.e. 40.3%) out of the 144 available.

Other random temporal sampling studies from our lab also showed systematically that the passage of time since target onset was not the only factor affecting processing efficiency. Indeed, time–frequency CIs demonstrated very strong effects of the predominant SNR oscillation frequencies within the stimulus which interact in complex ways with the time dimension. This general rule is verified in the present study, as shown in Fig. 4. Thus, for each SF condition, we find a unique pattern of processing efficiency that is jointly affected by time and the oscillation frequencies within the stimulus.

Again, we used an SVM classifier which was exposed to features from these time–frequency CIs with the task to determine the SF condition from which they originate. Classification performance reached 51.6% correct while using 79 features out of the 264 available (29.9%). This exercise was next repeated using features from the Fourier transforms of the time–frequency CIs (i.e. combined outcomes of the Fourier analysis of each stimulus oscillation frequency as a function of time). Remarkably, the accuracy of the SVM classifier was 90.6% correct (significantly superior to the 25% chance performance; binomial test *p* < 0.001) while using only 127 of the 1584 available features (i.e. 8.0%). The characteristic features for each SF condition which were extracted from the Fourier transformed time–frequency CIs are shown in Fig. 5.

## Discussion

The technique of random temporal sampling was used in the context of a word recognition task to examine how processing efficiency varies across exposure duration according to four conditions of bandpass SF filtering.

A first important aspect of the results is that the medium SF conditions (i.e. conditions 2 and 3) required about 2/3 of the target contrast of the more extreme SF conditions (i.e. conditions 1 and 4) to lead to identical levels of response accuracy (Table 1). Those intermediate SF conditions, with center frequencies of 2.4 and 4.8 cpd respectively, are in the 2 to 6 cpd range that is known as optimal for visual word recognition (see “Introduction”). If expressed in terms of object spatial frequency, these intermediate SF conditions have center frequencies of 3.5 and 7.1 cpl, respectively. The first condition matches the 2–4 cpl range known as optimal for single letter recognition (see Introduction) whereas the second is a bit higher. Overall, it may be concluded that the present study replicates the basic phenomenon of middle-range spatial frequencies being the dominant source of information in reading tasks.

**Figure 1:**
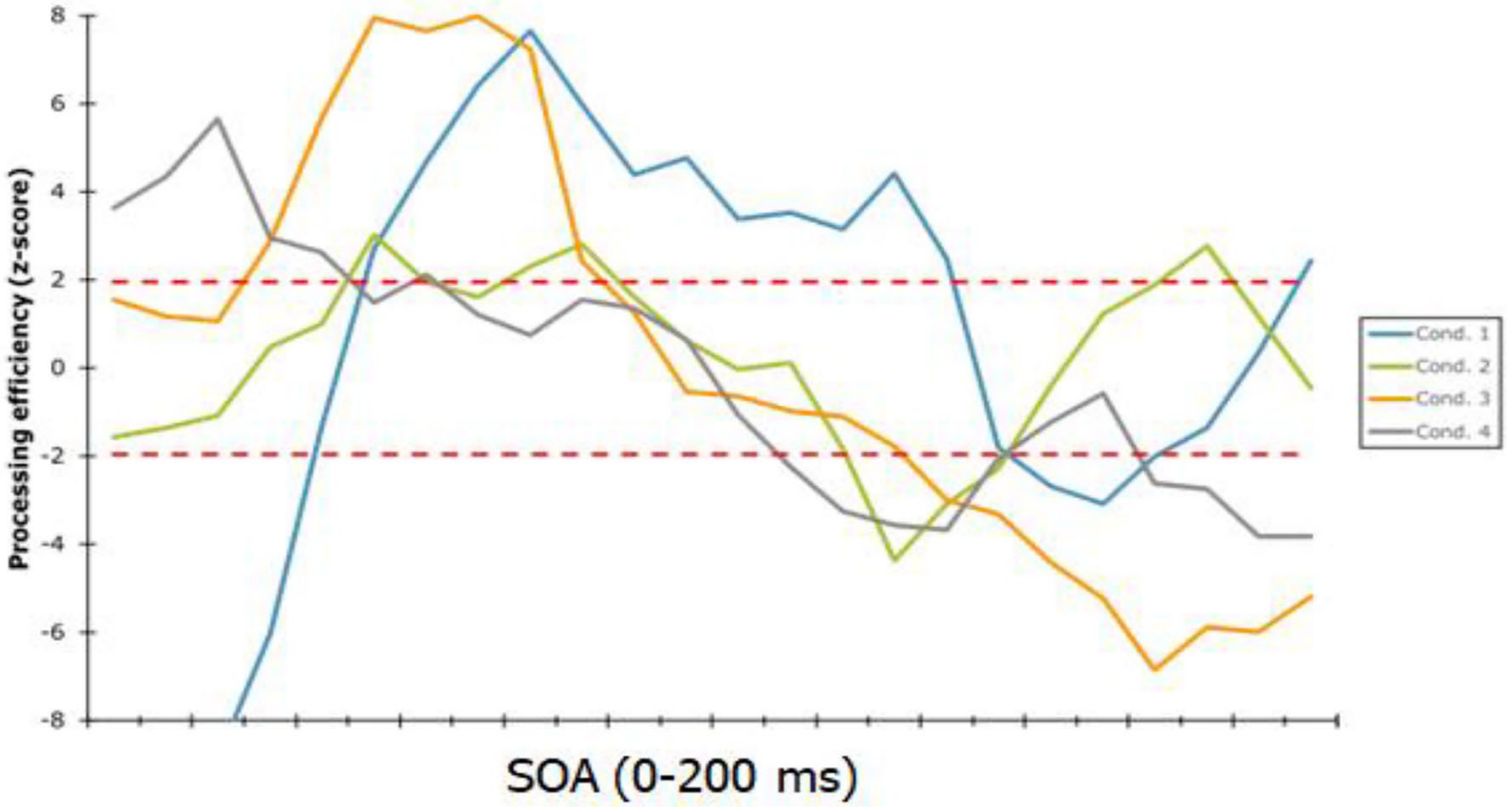
CIs of processing efficiency as a function of time for each SF condition. The two horizontal dashed red lines indicate the significance criterion which was determined by the Pixel test^29^. All portions of a curve above the upper threshold indicate a processing efficiency significantly superior to zero whereas those below the lower threshold indicate a processing efficiency significantly below zero.

One main outcome of the experiment with respect to the coarse-to-fine visual recognition theory are the time-domain CIs illustrated in Fig. 1. Excluding a potential interaction with the optimal SF range for word recognition, this theory predicted low SFs to dominate early processing, then followed by higher SFs. However, the findings from the time-domain CIs rather suggest an opposite SF order. Thus, upon target onset, it was the highest SF range that showed the greatest processing efficiency, then followed by the second highest SF range and then by the lowest of all.

**Figure 2:**
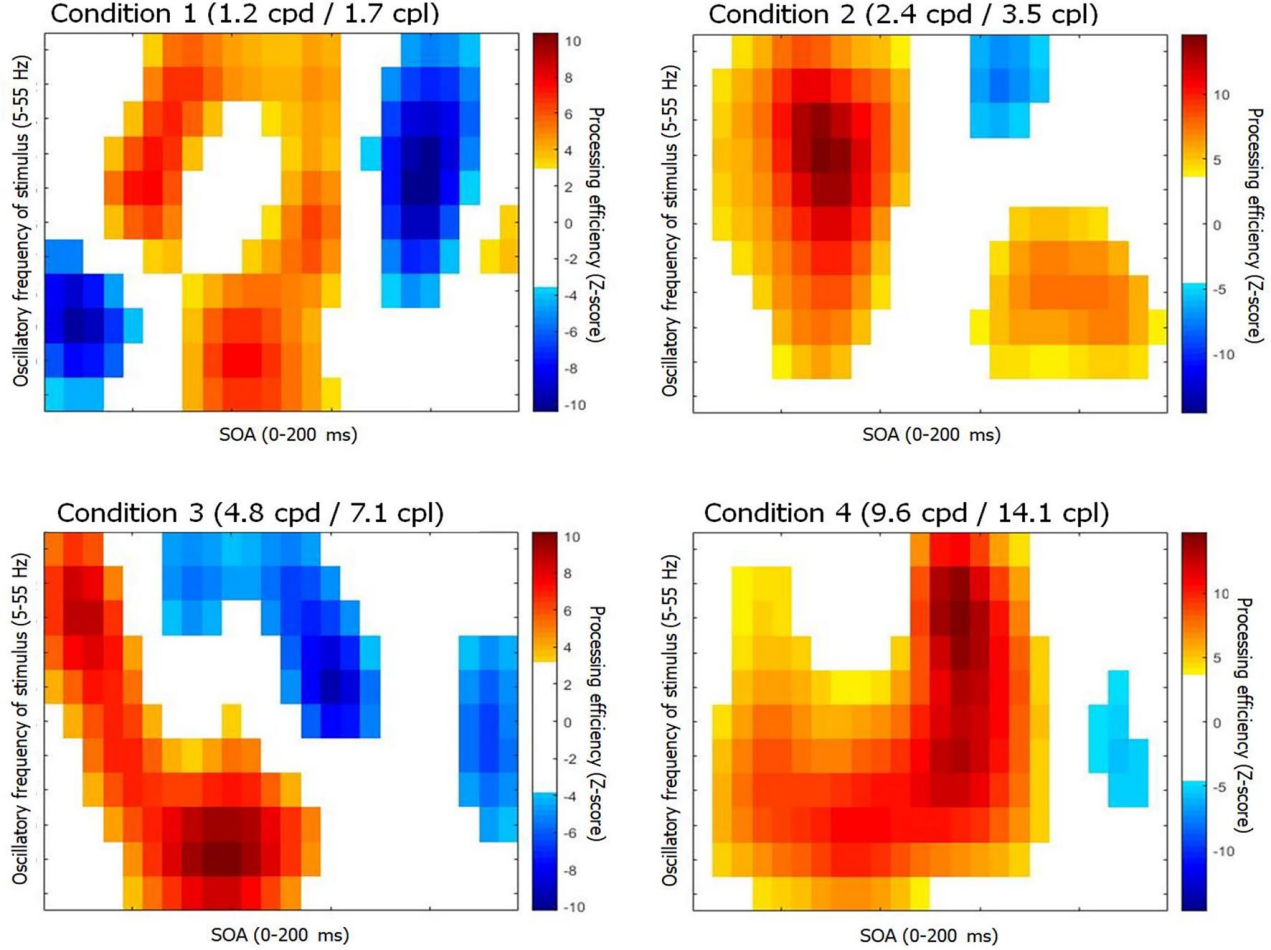
CIs of processing efficiency as a function of time and stimulus oscillation frequencies for each SF condition. Points in the CIs that are colored white do not differ significantly from zero, as determined by the Pixel test^29^.

Upon a deeper examination of the data, it became obvious that the time-domain CIs capture an incomplete story. Thus, the results show that processing efficiency for the different SF conditions is affected not only by the passage of time but also by the temporal frequency content of the SNR oscillations which control target visibility. This is evident from Fig. 2, which shows the time–frequency CIs for each SF condition. As far as visual word recognition is concerned, these findings indicate that the notion of a temporal order in spatial frequency processing proposed by the coarse-to-fine theory does not grasp the full breadth of the phenomenon under study. At present, the aspect of visual processing affected by the temporal frequency content of the SNR oscillations remains unknown. Regardless of what this may be however, the present findings clearly show that it is connected to way the visual system carries out the processing of spatial frequencies in a reading task.

Whether the complex interaction documented here between the frequency content of SNR oscillations, spatial frequencies, and time also occurs for other stimulus classes such as objects, scenes, or faces, is presently unknown. Thus, most of the previous studies investigating these stimulus classes have, in some way or another, manipulated the availability of spatial frequencies through time without jointly examining the impact of the temporal frequency content of such changes. Under the assumption that the processing of spatial frequencies occurs very early in the cortical visual pathways, one might be tempted to predict that the type of interaction documented here should be replicated with others stimulus classes. However, the dominance of intermediate spatial frequencies in visual word recognition seems to reflect the joint effects of two fundamental factors; i.e. the human contrast sensitivity function on the one hand and the spatial frequency range that best discriminates among letters on the other^4^. If this is so, and that the most discriminant spatial frequencies for others stimulus classes are different, then phenomena pertaining to spatial frequency processing should vary accordingly.

The available observations seem to militate in favor of the latter view. Thus, as noted above, if the passage of time is the only factor taken into consideration, the present observations suggest a fine-to-coarse processing order (i.e. high spatial frequencies first, followed by lower ones). In contrast, studies investigating other stimulus classes rather point to a coarse-to-fine processing order. This clearly implies that the way spatial frequencies are processed is strongly determined by the type of stimulus used. The word recognition study reported by Ref.^28^, (see “Introduction”) also supports this view by producing evidence incongruent with the notion of coarse-to-fine processing.

**Figure 3:**
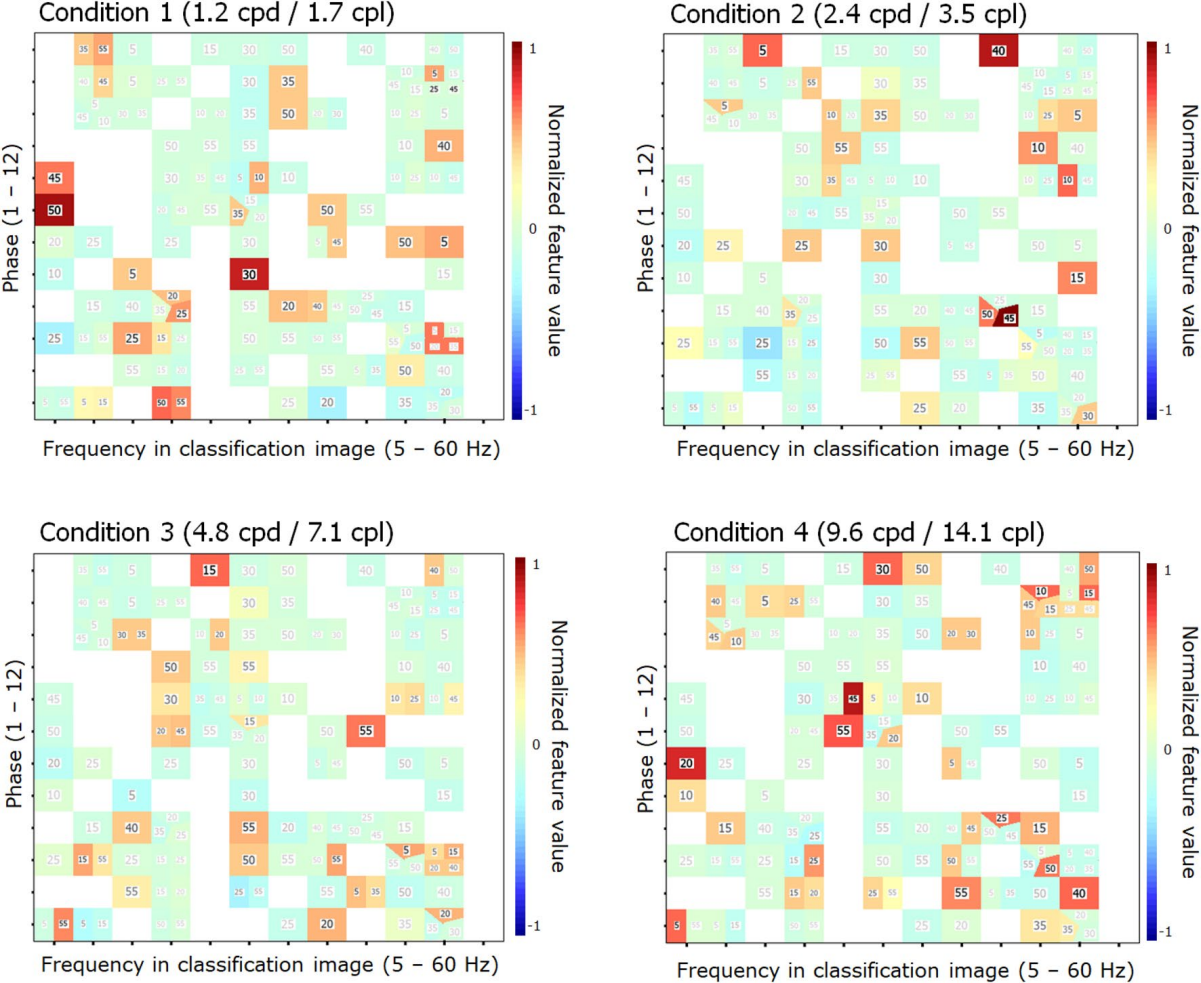
The temporal features characterizing each SF condition. These features are those which supported the 90.6% accuracy of the SVM classifier in categorizing the Fourier transforms of the time–frequency CIs of individual participants according to SF condition. The horizontal axis corresponds to the temporal frequencies extracted from the CIs, the vertical axis reflects the phase values of the extracted components and the colour code serves to illustrate the corresponding power values which have been normalized in the range – 1 to 1 (see description of methods for details). The numbers in the coloured patches indicate the frequency of target visibility oscillations that produced the Fourier features illustrated. The reader may zoom in to make them more readable. Phase x frequency cells may be occupied by more than one colour patch in cases where the Fourier analysis of the CI produced two or more features contributing to the SVM with the same phase x frequency combination but which came from different stimulation oscillation frequencies.

Another important observation from the present study is that the CIs of individual participants contain highly potent information regarding the spatial frequency band of the stimulus they are exposed to. Thus, the classification of individual data patterns according to SF condition by an SVM machine learning algorithm was well above chance regardless of the data format. In other words, this classification performance demonstrates a spatial frequency-specific signature within the CIs of individual participants. This signature within the IC frequency by SNR frequency domain is illustrated in Fig. 3.

What is striking in this regard, is the exceptionally high level of accuracy that was attained while using features from the Fourier transformed time–frequency CIs. Thus, using this data format, the SVM classifier reached 90.3% accuracy with only 8.0% of the total set of features available. This contrasts with the performance levels of about 40–50% correct achieved with the other data formats. Other random temporal sampling studies from our laboratory also produced similar findings. Clearly, the way the information content of CIs is coded may crucially affect the saliency of the spatial frequency-specific signature they contain.

It should not be surprising that the SVM classifier was more successful with the time–frequency than the time-domain CIs. Indeed, the former capture an aspect of the stimulation which interacted with the spatial frequency content of stimulation whereas the latter is blind to this information. What may seem shocking however, is the enormous gain in classification accuracy that is offered by the Fourier transforms of the time–frequency CIs compared to their untransformed versions. After all, their information content is identical and one can be transformed into the other with zero loss. A similar phenomenon was also reported by Arguin and Fortier-St-Pierre^1^ and Lévesque & Arguin^31^. What we believe is at play here is that transforming time–frequency classification images into their Fourier parameters eliminates the time dimension, which becomes recoded as a collection of phase and power values for several ranges of temporal frequency (i.e. 5–55 Hz in 5 Hz steps). It appears that this recoding may offer the CIs that are to be classified a better alignment to the brain mechanisms tapped by the task. We suggest that it will be important to investigate this issue in future studies.

## Conclusion

This study investigated the processing of spatial frequencies through time in a visual word recognition task using the paradigm of random temporal sampling. The main results point to a processing order going from high to low spatial frequencies. This is in contradiction to the theory of coarse-to-fine processing for visual recognition which had been supported by previous studies using other stimulus classes. Also important, our findings indicate that the temporal frequency content of the stimulus has a major impact on the processing of spatial frequencies which interacts with the passage of time. We propose that this complex interaction needs to be investigated further in future studies to offer a better grasp of how the human visual system uses spatial frequencies for the purpose of recognition.

## Methods

### Participants

Sixteen adults (4 men and 12 women; mean age of 21.8 y.o) with normal or corrected vision and normal neurological function were recruited for this experiment. All participants gave their informed consent and the experimental protocol was approved by the Comité d’Éthique de la Recherche en Éducation et Psychologie of the University of Montreal. All methods were performed in accordance with the relevant guidelines and regulations. Participants each received 60$ as compensation.

### Materials and stimuli

**Figure 4:**
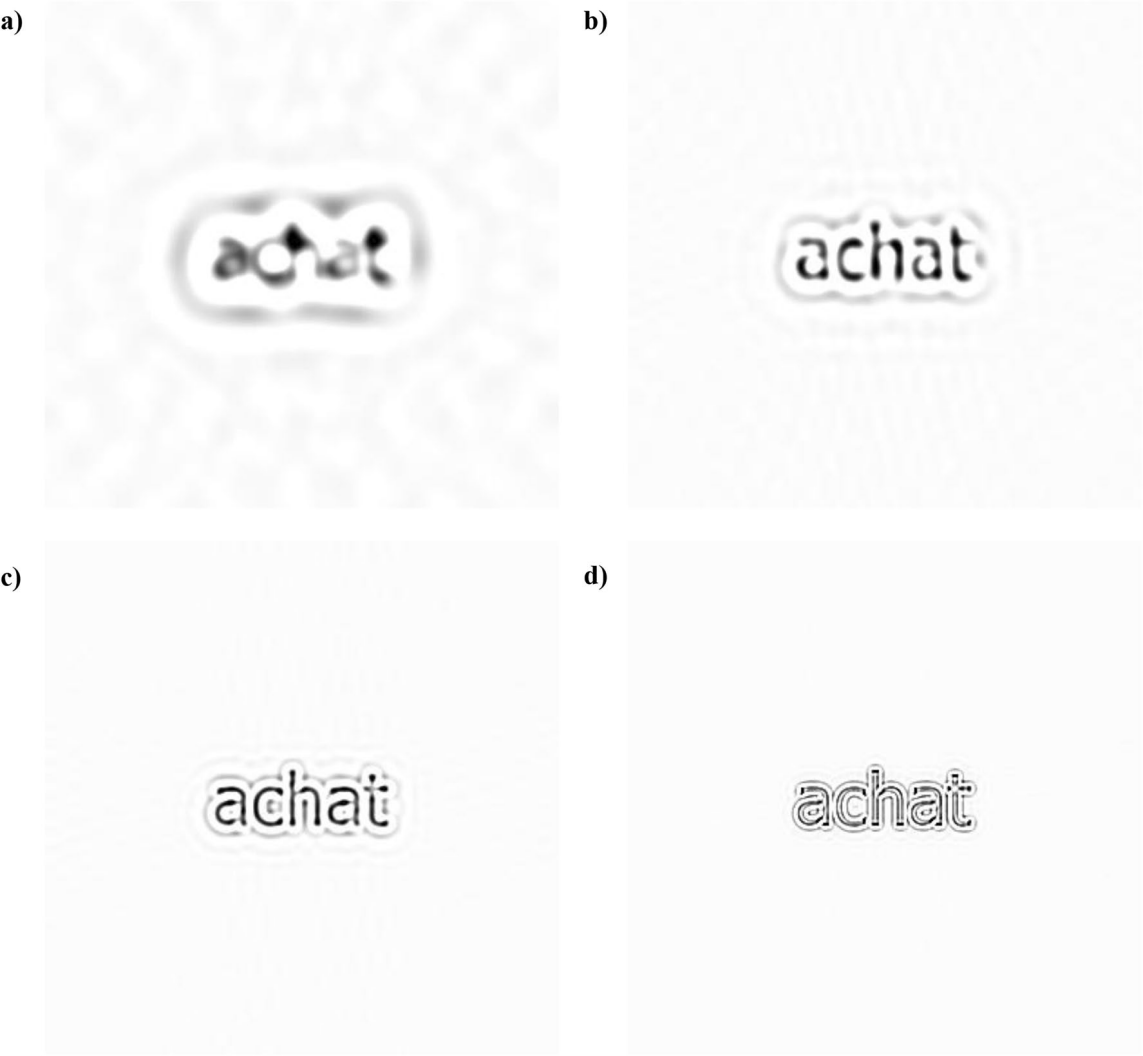
Each panel illustrates the French word “achat” spatially filtered according to each SF condition: (**a**) 1.2 cpd/1.7 cpl, (**b**) 2.4 cpd/3.5 cpl, (**c**) 4.8 cpd/7.1 cpl, (**d**) 9.6 cpd/14.1 cpl. In the present images, the SF content is obviously function of observation distance.

The experiment was programmed in MATLAB (©1994-2017, The MathWorks Inc.) and made use of functions from the Psychophysics toolbox^32^ for stimulus display. It was run on an ASUS VG248QR HD monitor with maximum luminance of 200 cd/m^2^ and a 120 Hz refresh rate. All manipulations of pixel luminance were linear. The observation distance of the participants was 57 cm with their heads supported by a chin rest. Stimuli were 424 five-letter French common words printed black on white in Tahoma font. Their horizontal spatial extent was of 3.4 deg and x-height was of 0.75 deg. Word images were filtered according to four SF conditions using a bandpass Butterworth filter (Fig. 4). The center frequencies and cutoffs (in parentheses for each condition) were 1.2 (0.9–1.5), 2.4 (1.8–3.0), 4.8 (3.6–6.0), and 9.6 (7.2–12) cpd (in object spatial frequency metric: 1.7 (1.3–2.2), 3.5 (2.6–4.4), 7.1 (5.3–8.8), and 14.1 (10.6–17.6) cycles per letter – cpl).

**Figure 5:**
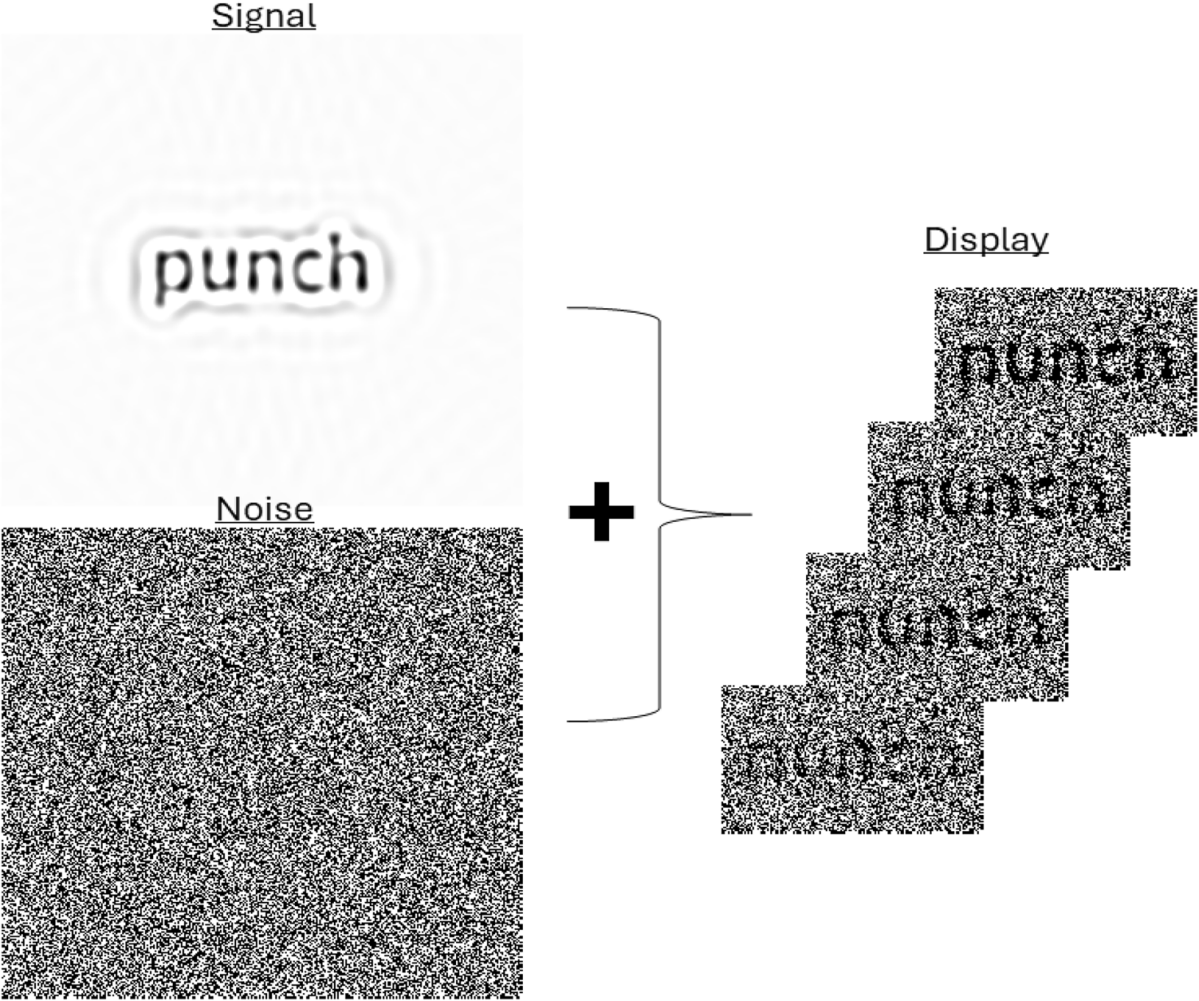
Illustration of the composition of the stimuli displayed on each trial, which were made of an additive combination of the target image and of a white noise field. In the present example, the signal was the spatially filtered word “punch”. Below is the white noise field with which it was combined to produce the ‘Display’ portion of the figure. This latter panel shows four different levels of signal-to-noise ratio, from high to low as one moves downwards. On each trial, the signal and noise portions of the stimuli remained constant but the signal-to-noise ratio varied randomly throughout exposure duration.

The stimuli were a linear summation of the signal (i.e. spatially filtered target word) and the noise, which was an overlaid white noise field (Fig. 5) and the stimulation area of 8.9 × 8.9 deg was displayed over a black background. These stimuli were presented for a duration of 200 ms through which the SNR varied according to a random sampling function made by the integration of sine waves with frequencies ranging between 5 and 55 Hz in steps of 5 Hz with random amplitudes and phases. This SNR function was then normalized in the range 0 to 0.75. Thus, the visibility of the target word among the noise varied randomly throughout its exposure duration. New, independent SNR functions and white noise fields were generated for each trial.

### Procedure

Prior to the experimental phase, participants went through a practice round that consisted in 24 five-letter French words (different from those used in the experimental phase) that were spatially filtered according to the SF conditions described above (6 trials per condition). For the experimental phase, participants went through four blocks of 200 trials each (i.e. 800 trials) per session, for a total of 3200 trials over four sessions. No target word was repeated in a block and each word was presented a total of eight times in the experiment, that is twice for each SF condition.

The time course of each trial was as follows: 1- onset of white noise field without target for a duration of 1000 ms; 2- a white fixation cross (1.1 × 1.1 deg) was then added over the white noise field for 250 ms and then withdrawn; 3- in the 250 ms time interval between the offset of the fixation cross and the onset of the target, a 900 Hz, 75 dB tone of 50 ms duration was presented; 4- target display lasting 200 ms which was made of a sequence of 24 images with randomly varying SNR (see “Materials and stimuli”) at a 120 Hz rate; 5- onset of four response choices displayed in normal (i.e. unfiltered) print above, below, to the right and the left of the target display area. The response choices remained visible until the participant indicated which he/she thought was the target by pressing the up, down, right or left arrow key on the computer keyboard. The computer emitted a high-pitch tone (1000 Hz, 100 ms) following a correct response and a low-pitch one (300 Hz, 300 ms) following an error. The response was followed by a 1000 ms delay before the following trial was initiated.

The three distractor images for the four-alternative forced-choice task (4AFC) were selected among the list of experimental words (excluding the target) based on their image cross-correlation (used here as a proxy for visual similarity) with the target. The distractor selection algorithm was applied at the beginning of the experiment and preset the distractors to be used on every experimental trial. Its goal was to maximize the visual similarity between distractors and the target while maintaining an overall difficulty level (i.e. summed image cross-correlations with the target for the three distractors) as equal as possible across all trials. A final constraint was that no word could be used as distractor for more than 6 different targets.

The performance of participants in the experimental phase was maintained at about 50% accuracy for each condition by using a staircase procedure manipulating the contrast level of the signal (i.e. the image of target word) portion of stimuli. Target contrast was of 35% in the practice phase and remained thus at the beginning of the experimental phase. Following the first 20 experimental trials, response accuracy for the preceding 10 trials of the condition tested on the last trial was assessed. If accuracy was above 50% correct, target contrast for that condition was decreased by one step whereas it was increased by one step when accuracy was below 50%. The initial step size for a change of target contrast was of 16%. After every reversal in the direction of adjustments for a condition, this step was halved, down to a minimum of 1%. The state of this contrast adjustment algorithm was maintained across consecutive experimental blocks.

Each test session lasted about 60 min, for a total duration of the experiment of four hours per participant.

### Data analysis

Time-domain CIs were calculated for each participant and each SF condition by doing a weighted subtraction of the SNR sampling functions of experimental trials associated with errors from those associated with correct responses. These CIs reflect processing efficiency as a function of the time elapsed from target onset to its offset. The notion of processing efficiency is not to be confused with actual performance. It rather reflects the capacity of a participant to use whatever information is available from the stimulus (here, at a particular time) to reach a correct response.

As will be shown in the “Results” section, the pattern of results reflected by the time-domain CIs was quite different from our expectations. This led us to a deeper examination of the results by considering not only time as the single factor potentially affecting processing efficiency, but also the oscillatory power of the SNR sampling functions along a range of frequencies. This was done by calculating CIs based on time–frequency representations of the SNR sampling functions. Thus, the SNR sampling functions of each individual trial were submitted to a wavelet analysis using three-cycle complex Morlet wavelets varying in temporal frequency from 5 to 55 Hz in 5 Hz steps. The number of cycles selected for the Morlet wavelet was aimed at obtaining a high precision along the time dimension, while somewhat sacrificing precision on the frequency dimension. As for the time-domain CIs, those in the time–frequency domain were calculated by the weighted subtraction of sampling functions associated with errors from those associated with accurate responses.

Individual raw CIs were transformed into Z scores by a bootstrapping operation^33^. Once in a common scale, the CIs of individual participants were averaged and then smoothed using a Gaussian filter. For time-domain CIs, this filter had a full width at half maximum (FWHM) of 19.6 ms. For the time–frequency CIs, the filter had a FWHM of 29.3 ms on the time dimension and of 17.7 Hz on the frequency dimension. The filtered CIs were then submitted to a two-way Pixel test^29^ with α = 0.05 to determine which points in the images differed significantly from zero.

The assessment of the distinctiveness of data patterns across conditions was done using the classification performance of a linear support vector machine (SVM^34^) along with a leave-one-out cross-validation procedure applied to either the CIs themselves or their Fourier transforms. To learn the mapping from a particular data pattern format to the corresponding SF condition, the SVM was given a subset of the available features from all the individual CIs (or their Fourier transforms) but one. Then, the SVM had to determine the condition corresponding to the data pattern that had been left out of the learning phase. This was repeated by leaving out a different CI (or its Fourier transform) until we had iterated through all of them. The percentage of iterations on which the SVM reached a correct response was used to determine classification accuracy. Binomial analyses assessed if classification accuracy deviated significantly from chance.

The classification of data patterns using an SVM satisfied several important aims. The most obvious is that an accuracy that is greater than chance implies that there exist important (i.e. significant) differences in the data patterns that are contrasted. Of course, standard statistical procedures can do so as well. However, less obvious but crucially important is that it also provides an indication that these data patterns are replicable across individuals. Indeed, even if average data patterns are markedly different across the conditions compared, if they are not replicable across individuals, the performance of the classifier will be poor. In other words, to obtain a highly accurate classifier, the relevant features in the training patterns must retain their value in the pattern that is used in the test phase. This feature of an SVM classifier is particularly important here since the Pixel test used to determine whether individual points in CIs differ from zero ignores individual differences in data patterns. Finally, an added bonus of using a classifier is that we can determine the features in the data patterns from which its discriminatory power is derived. This thus permits to specify the feature values that characterise each SF condition.

In order to retain only the most relevant features that discriminate among conditions, we used a stepwise procedure for the introduction of features to the model one at a time, in a way that resembles the technique of stepwise multiple regression. In an initial step, only the most valuable feature was offered to the SVM. The next best feature was then added to the model on the following step, and so on. The procedure was interrupted when either the SVM achieved a classification performance of 90% correct or when all the available features had been presented, whichever occurred first.

The order in which CI features were introduced to the SVM model was based on the capacity of each possible feature to discriminate among the SF conditions. This index of discrimination capacity was analogous to an F ratio; i.e. it was measured by the ratio of the variance of the means across conditions over the error variance. Thus, the feature with the greatest discrimination index was entered first, followed by the second greatest, and so on, until the stopping criterion was reached.

For the illustration of the characteristic features of each SF condition, the data retained was that pertaining to the features used at the point when the stopping criterion was reached. The representation of a feature at each SF condition was based on the squared difference between its mean and the overall mean across conditions, which was divided by the error variance (see above). These values were then linearly normalized in the range -1 to 1 based upon the maximum absolute value among the full set of features to illustrate across all conditions. To facilitate focussing on the strongest levers for classification, i.e. the features with the most extreme values, the contrast of the color code used to illustrate feature values was linearly diminished according to their distance from the extremes of the scale (i.e. − 1 or 1), down to a minimum of 30% (to maintain visibility of even the weakest features illustrated). However, when the value of a feature for a particular condition was exactly 0, it was omitted from the figures.

Repeated measures analyses of variance (ANOVAs) were conducted to compare the SF conditions on response accuracy as well as on the average level of contrast under which these performances were attained.

## Data Availability

The raw data from the present study will be made available upon request to MA (martin.arguin@umontreal.ca). We will be placing this data in an open access repository in the near future.
